# M3S: a comprehensive model selection for multi-modal single-cell RNA sequencing data

**DOI:** 10.1186/s12859-019-3243-1

**Published:** 2019-12-20

**Authors:** Yu Zhang, Changlin Wan, Pengcheng Wang, Wennan Chang, Yan Huo, Jian Chen, Qin Ma, Sha Cao, Chi Zhang

**Affiliations:** 1 MOE Key Laboratory of Symbolic Computation and Knowledge Engineering, Colleges of Computer Science and Technology, Jilin University, Changchun, 130012 China; 2 Center for Computational Biology and Bioinformatics, Indiana University, School of Medicine, Indianapolis, 46202 IN USA; 3 Department of Electronic Computer Engineering, Purdue University, West Lafayette, IN 47907 USA; 4 Department of Computer Science, Indiana University-Purdue University Indianapolis, Indianapolis, IN 46202 USA; 5 School of Fundamental Sciences, China Medical University, Shenyang, 110122 China; 6 Shanghai Pulmonary Hospital, Tongji University School of Medicine, Shanghai, 200082 China; 7 Department of Biomedical Informatics, The Ohio State University, Columbus, OH 43210 USA; 8 Department of Biostatistics, Indiana University, School of Medicine, Indianapolis, 46202 IN USA; 9 Department of Medical and Molecular Genetics, Indianapolis, IN 46202 USA

**Keywords:** Single cell RNA-seq, Multimodality, Differential gene expression analysis, Drop-seq, Left truncated mixture Gaussian

## Abstract

**Background:**

Various statistical models have been developed to model the single cell RNA-seq expression profiles, capture its multimodality, and conduct differential gene expression test. However, for expression data generated by different experimental design and platforms, there is currently lack of capability to determine the most proper statistical model.

**Results:**

We developed an R package, namely Multi-Modal Model Selection (M3S), for gene-wise selection of the most proper multi-modality statistical model and downstream analysis, useful in a single-cell or large scale bulk tissue transcriptomic data. M3S is featured with (1) gene-wise selection of the most parsimonious model among 11 most commonly utilized ones, that can best fit the expression distribution of the gene, (2) parameter estimation of a selected model, and (3) differential gene expression test based on the selected model.

**Conclusion:**

A comprehensive evaluation suggested that M3S can accurately capture the multimodality on simulated and real single cell data. An open source package and is available through GitHub at https://github.com/zy26/M3S.

## Background

A large number of single-cell RNA sequencing (scRNA-seq) data sets have been recently generated to characterize the heterogeneous cell types or cell states in a complex tissue or biological process [1–5]. Gene expression in a single cell is purely determined by the transcriptional regulatory signal in the current cell, which may vary drastically throughout different cells. Hence, a gene’s expression could display multiple regulatory states across multiple cells, that naturally form a multi-modal distribution, where each modality corresponds to a potential regulatory state [6]. Many statistical models have been developed to model gene expressions for cells collected under different conditions or data generated by different experimental platforms, including Poisson (P), Negative Binomial (NB), Gausian (G), Zero Inflated Poisson (ZIP), Zero Inflated Negative Binomial (ZINB), Zero Inflated Gaussian (ZIG), Mixture Gaussian (MG), Beta Poisson (BP), Zero Inflated Mixture Gaussian (ZIMG), Left Truncated Gaussian (LTG) and Left Truncated Mixture Gaussian (LTMG) distributions, among which some are designed to capture expression multi-modalities. In addition to the multi-modality assumptions, these models also differ by their assumptions used to model “drop-out” events, and error distributions [6–11]. We have recently developed a systems biological model to interpret the biological underpinnings of multi-modality, drop-outs and other errors in a scRNA-seq data. Our analysis and other recent works clearly suggested that experimental condition and platform bias should be considered while we select the best model to fit scRNA-Seq data, as they largely contribute to the variabilities of interest [12]. However, there is lack of a computational tool in the public domain for a proper model selection in a scRNA-seq data set and downstream differential gene expression analysis based on multi-modality model assumption.

Motivated by this, we developed a user-friendly R package, M3S, to (1) select the most proper statistical models and differential gene expression test method, (2) characterize varied transcriptional regulatory state, and (3) detect differentially expressed genes among given conditions, for scRNA-seq data. The tool can be generalized to bulk tissue transcriptomics or other omics data if considering multi-modality is necessary. The M3S package is available at: https://github.com/zy26/M3S.

### Implementations

M3S package imports two additional packages, “mclust” and “pscl”, for fitting of a MG model and estimating parameters of a ZINB model, respectively [13, 14]. For information on the latest versions of imported packages and functions, see the package’s DESCRIPTION and NAMESPACE files (https://github.com/zy26/M3S**).** An S4 class is used to store numerical properties of the input gene expression data. *M3S* is the main function, which implements model selection for each gene, and outputs a list contains the estimated parameters, model fitness, and *p* values of the goodness of fitting, given each candidate model. We have adopted a dynamic function call model approach so that future extensions will be convenient.

**Fig. 1:**
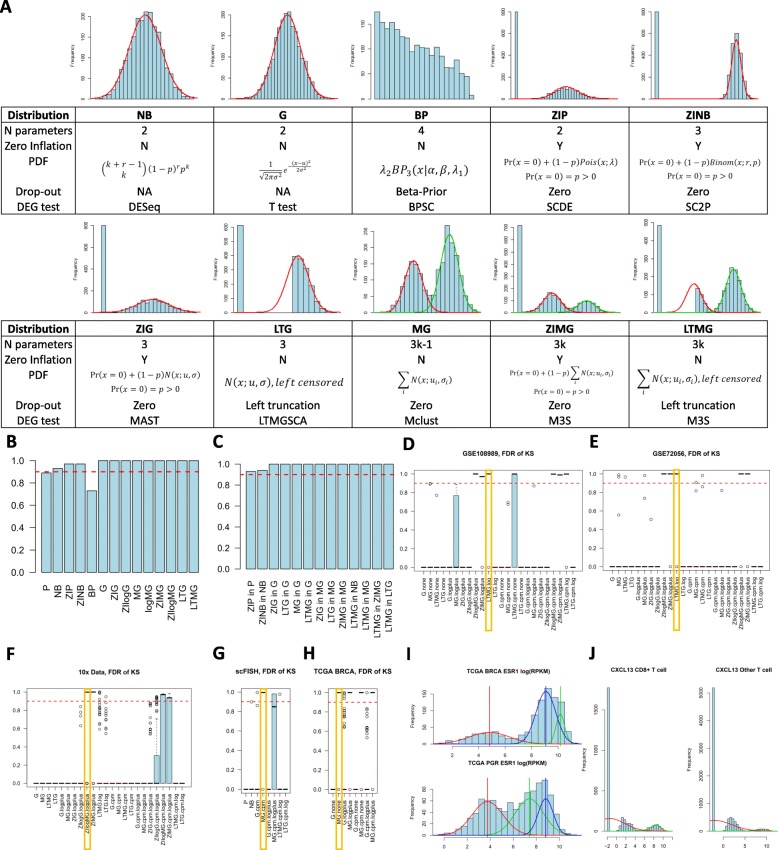
**a** Details of considered distributions; **b** Rate of the simulated features that can be corrected predicted by *M3S*; **c** Rate of the simulated outliers that can be corrected identified by *M3S*. The x-axis represents the distribution of the outlier in the simulated data of a specific distribution. **d-h** Boxplots of FDRs of the fitting by selected distributions on 100 selected features of the GSE108989 (**d**), GSE72056 (**e**), 10x (**f**), scFISH (**g**), and TCGA BRCA (**h**) data. The selected best model is highlighted. **i** Gene expression profile of ESR1 and PGR in TCGA BRCA samples. **j** Gene expression profile of selected gene show a differential gene expression in high expression peak between CD8 + T cell and other T cells in the GSE108989 data set

The core function *M3S* can be directly exported from the M3S package. The input of this function is a gene expression data matrix, where rows indicate genes/transcripts and columns indicate samples. The output is organized into a list, each element of which includes an indication of the most proper model relating to each gene/transcript feature in the expression matrix, as well as the complete fitting statistics of all examined models. Specifically, the *M3S* function first assesses several data characteristics by checking if the data is (1) nonnegative (2) with significant proportion of zero observations, (3) discretized, and (4) with negative infinite observations. Then based on the data characteristics, *M3S* provides data specific normalizations among (1) log, (2) log(X + 1), (3) CPM, (4) log (CPM), and (5) log (CPM + 1) transformations. After normalization, M3S fits each row with the selected models that can fit the data type, and selects the best one. M3S defines the best model as the most parsimonious one that significantly fits the observed expression distribution by using a Kolmogorov Simonov Statistics (see details in Additional file 1: Figure S1. Supplementary Note). We consider the models complexity is ordered as *P* < NB, G < ZIP < ZINB, ZIG, LTG < BP < MG < ZIMG, LTMG (Fig. 1a). Due to the unfixed number of model parameters, the complexity between, MG, ZIMG and LTMG will be selected if the number of peak of one of the distribution is significantly smaller than the number of peaks fitted by the others, by using a Mann Whitney test.

In addition, the M3S package offers the fitting parameters of the best fitted model and gives the most proper data normalization and differential gene expression test method for the input data set. The *M3S.fit* function enables parameter estimations for a given model. The *M3S.test* function identifies differentially expressed genes by hypergeometric test, and in detail, by testing whether samples falling under one peak of the multi-modal distribution significantly enriches pre-specified sample collections (See more details in the Additional file 1: Figure S1. Supplementary Note).

## Results

### Validation of M3S on simulation data

We benchmarked the M3S package on simulated data sets and four real scRNA-seq data sets. We first simulated data sets composed by features of the 11 selected distributions. For the simulation dataset, 100 features (random variable) were simulated on 500 samples from one of the 11 distributions. The simplest model that is with FDR of the Kolmogorov Simonov statistics larger than 0.1 is selected as the best model. We tested if M3S can accurately identify the corrected model distribution for each feature, and found out, M3S achieves a 96.35% accuracy (Fig. 1b). The only distribution that M3S achive less than an 85% accuracy is BP, majorly due to a bias lead by the Gauss-Jacobi quadrature approximation of the CDF of the BP model. We further added a few “noise” features, each of which has a distribution other than the true distributions specified. It turns out that M3S has high specificity and can effectively identify the outlier features with an over 98.5% accuracy on average (Fig. 1c).

### Application of M3S in detecting the multi-modality of expressions on real data sets

We further tested M3S on four real single cell data sets and one bulk tissue data, including (1) a T cell scRNA-seq dataset generated by SMART-seq2 platform, consisting of 11,138 cells (GSE108989) [15], (2) a scRNA-seq data set of 4645 stromal, immune and cells in melanoma micro-environment generated by C1/SMART-seq platform (GSE72056) [5], (3) a data set of PBSC generated by 10x genomics consisting of 4590 peripheral blood cells [4], and (4) a single cell FISH data set of 347 cells and 20 genes [16], and (5) TCGA breast cancer (BRCA) RNA-seqV2 data containing 1091 breast cancer tissue samples [17]. These datasets cover three platforms for single cell expression and one for bulk tissue expression profiling that are most popular. Our analysis suggested that in general, LTMG is the best model for log transformed CPM data generated by C1/SMART-seq and SMART-seq2 platforms; ZIMG is the best model for the log transformed CPM data the generated by 10x genomics, and the MG is best for modeling log normalized data generated by single cell FISH and the TCGA-BRCA data (Fig. 1d-h). These could be explained by the distinctions of different technologies used to profile and collect the data: (1) reads data generated under the C1/SMART-seq and SMART-seq2 platforms are often saturated, meaning there exists a minimal expression level representing a common experimental resolution for all samples, hence truncating the gene expression below the experimental resolution as in LTMG is rational; (2) reads data generated by 10x genomics are, however, always unsaturated, and the experimental resolutions are highly varied through cells, thus handing the varied experimental resolutions with Gaussian errors as in ZIMG performs better in fitting the data comparing to LTMG; (3) scFISH data are with multi-modality but a small amount of zero observations.

It is noteworthy that 55 and 37% of the genes in the (tested) SMART-seq/SMART-seq2 and 10x data have more than one (non-zero) peaks, suggesting the necessity of considering multi-modality in the single cell expression data modeling. In the TCGA BRCA data, our model identified that around 31.9% genes were best fitted by either the MG or LTMG model with more than one peaks, such as the ESR1 and PGR genes that are associated with the breast cancer subtype (Fig. 1i). We also evaluated the computational efficiency of M3S, and our analysis suggests that M3S can select and fit the best model for 100 features of 1000, 5000, and 10,000 real single cell samples in 618 s, 1022s and 7255 s, by using a PC with an Intel Core i7-7700K CPU (4.20 GHz) and 16G RAM.

### Application of M3S on differential gene expression test for simulated and real scRNA-seq data sets

We applied the *M3S.test* function to identify differentially expressed genes associated with pre-defined sample classes in the T cell scRNA-seq data set. We compared M3S with MAST, which is currently one of the most commonly used differential gene expression analysis method for scRNA-seq [8]. One of our results clearly suggests that 160 genes are with more than one non-zero peak are significantly associated with CD8+ T cells (identified by using *M3S.test*, FDR < 0.05), as illustrated in Fig. 1j.

## Discussion

M3S is developed for gene-wise model selection, and particularly, comprehensive inference of the modality of individual gene’s expression in a scRNA-seq data. On 20 sets of single cell RNA-seq data generated by Smart-Seq/Smart-Seq2 protocols, we discovered that LTMG represents the best model for majority of the genes [6]. On the other hand, for the drop-seq based scRNA-seq data, such as 10x genomics platform, the experiment resolution are varied throughout different cells as with the total captured counts. Our analysis suggests that ZIMG achieved best fitting for 10x genomics data sets. Considering the error of the lowly (non-zero) expressions are hard to be modeled due to the varied experiment resolutions, ZIMG model utilizes a Gaussian distribution to cover the variation of the errors of the lowly expressed genes. For a gene fitted with multiple peaks in a drop-seq data set, we suggest considering the zero expressions as well as those expressions falling into the lowest peak as insignificant expressions, while the rest of the expressions in larger peaks as different levels of true expressions.

Noting that the gene expression in a single cell is purely determined by the sum of current transcriptional regulatory inputs in the cell, the multi-modality of a single gene’s expression may suggest heterogenous transcriptional regulatory states of the gene throughout different cells. A group of genes consistently falling into a same peak throughout a certain subset of cells, would suggest that these genes may possibly be co-regulated by a transcriptional regulatory signal specifically in these cells. Hence identification of gene co-regulation modules can be mathematically formulated as finding submatrices, in which the expression of its pertinent genes on its containing samples are consistently classified to one certain peak of its multiple peaks. This can be solved by integrating *M3S* and *M3S.fit* functions with a bi-clustering detection algorithm [18, 19].

## Conclusion

Our comprehensive evaluation suggested the M3S package can accurately capture the multimodality on simulated and real single cell data. An open source package and is available through GitHub at https://github.com/zy26/M3S.

## Availability and requirements

Project name: M3S.

Project home page: https://github.com/zy26/M3S

Operating system(s): Platform independent.

Programming language: R.

Other requirements: R.3.5 and above.

Any restrictions to use by non-academics: license needed.

## Supplementary information

**Additional file 1: Figure S1.** Supplementary Note.

## Data Availability

All the codes and testing data were provided at https://github.com/zy26/M3S.

## Abbreviations

BP: Beta Poisson
BRCA: Breast Carcinoma
FISH: Fluorescent in Situ Hybridization
G: Gausian
LTG: Left Truncated Gaussian
LTMG: Left Truncated Mixture Gaussian
M3S: Multi-Modal Model Selection
MG: Mixture Gaussian
NB: Negative Binomial
P: Poisson
TCGA: The Cancer Genome Atlas
ZIG: Zero Inflated Gaussian
ZIMG: Zero Inflated Mixture Gaussian
ZINB: Zero Inflated Negative Binomial
ZIP: Zero Inflated Poisson
