# Supplementary material for: M3S: a comprehensive model selection for multi-modal single-cell RNA sequencing data
Source: BMC Bioinformatics. 2019 Dec 20;20(Suppl 24):672. doi: 10.1186/s12859-019-3243-1 (PMC6923906; doi:10.1186/s12859-019-3243-1)
Supplement: Supplementary file 1 — Additional file 1: Figure S1. Supplementary Note. [file 12859_2019_3243_MOESM1_ESM.pdf]

M3S: A comprehensive model selection for multi-modal single-cell RNA sequencing data.

Yu Zhang[1,2*+], Changlin Wan[2,3+], Pengcheng Wang[4], Wennan Chang[2,3], Yan Huo[2,5], Jian Chen[6], Qin Ma[7] Sha Cao[2,8], Chi Zhang[2,3,9*]

[1]Colleges of Computer Science and Technology, Jilin University, Changchun,130012, China,

[2]Center for Computational Biology and Bioinformatics, [8]Department of Biostatistics, Indiana University, School of Medicine, [9]Department of Medical and Molecular Genetics, Indianapolis, IN,46202, USA.

[3]Department of Electronic Computer Engineering, Purdue University

[4]Department of Computer Science, Indiana University-Purdue University Indianapolis, Indianapolis, IN,46202, USA.

[5]Department of Computer Science, China Medical University, Shen Yang, 110001, China

[6]Shanghai pulmonary hospital, Shanghai, China, 200082

[7]Department of Biomedical Informatics, the Ohio State University, Columbus, OH, 43210, USA,.

[+]The authors have equal contribution to this work.

*To whom correspondence should be addressed: +1 317-278-9625; Email: czhang87@iu.edu. Correspondence is also addressed to Yu Zhang: Email: zy26@jlu.edu.cn

Supplementary Notes

M3S function

M3S first identifies if a data is (1) nonnegative (2) with significant proportion of zero observations, (3) discretized, and (4) with negative infinite observations. The data is normalized by certain methods and fitted with different models based on its characteristics, as detailed below:

**Case 1**	
Nonnegative	True
With significant proportion of zero observations	True
Discretized	True
With negative infinite observations	False
Consider normalization	Data, CPM, log(Data), log(CPM), log(Data+1), log(CPM+1)
Correpsonded data type	Raw Counts of scRNA-seq
Considered models	P, NB, ZINB, ZIP: Data; BP, G, MG, ZIG, ZIMG, ZIlogG, ZilogMG: CPM, log(Data+1), log(CPM+1); LTMG, LTG: log(Data), log(CPM)

**Case 2**	
Nonnegative	True
With significant proportion of zero observations	False
Discretized	True

With negative infinite observations	False
Consider normalization	Data, CPM, log(Data), log(CPM)
Correpsonded data type	Raw Counts of bulk tissue RNA-seq data
Considered models	P, NB: Data; BP, G, MG: CPM, log(Data), log(CPM)

Case 3	
Nonnegative	True
With significant proportion of zero observations	True
Discretized	False
With negative infinite observations	False
Discretized	CPM, log(Data), log(CPM), log(Data+1), log(CPM+1)
Correpsonded data type	Normalized scRNA-seq
Considered models	BP, G, MG, ZIG, ZIMG, ZIlogG, ZilogMG: CPM, log(Data+1), log(CPM+1); LTMG, LTG: log(Data), log(CPM)

Case 4	
With significant proportion of zero observations	True
Discretized	False
With negative infinite observations	False
Discretized	False
Correpsonded data type	CPM, log(Data), log(CPM), log(Data+1), log(CPM+1)
Correpsonded data type	Normalized bulk tissue RNA-seq data & microarray data
Considered models	BP, G, MG, ZIG, ZIMG, ZIlogG, ZilogMG: CPM, log(Data+1), log(CPM+1), log(Data), log(CPM)

Other Cases	Wrong input data

If a data set is with more than 100 features, to save the running time the method will randomly draw 100 features from the data for the distribution selection.

Identification of the most proper statistical model for a given data set:

The models' complexity is ordered as P < NB, G < ZIP < ZINB, ZIG, LTG < BP < MG < ZIMG,

LTMG, and the MG, ZIMG and LTMG will be selected if the number of peaks of one of the distributions is significantly smaller than the number of peaks fitted by the others, by using a Mann Whitney test. The M3S will compute a KS statistic and corresponding p value for the fitting of each distribution. For each model, FDR corrected p values were used to evaluate if the model can fit the data well. Considering the data may contain a certain number of outliers, especially a certain rate of lowly expressed features, the default criteria of M3S for “a model with a significant fitting to the data” is set as “more than 90% of the features are with FDR>0.1 of the KS statistics of the model’s fitting”. This cut-off can also be modified by the users by changing the FDR_cutoff parameter. See more details at: https://github.com/zy26/M3S.

M3S.fit function

This function fit a data with a selected distribution. The input is a data matrix and pre-specified normalization method and distribution. The output is the fitting parameter. See more details at: https://github.com/zy26/M3S.

M3S.test function

This function test if the multi-modality of the selected distribution is significantly associated with a predefined cell class. The *M3S* function first determines the best fitting model and the *M3S.fit* function provides fitted parameters. We consider ZIP, ZINB, ZIG are bimodal models with zero as one model component and non-negative values as the other model component. MG, ZIMG, and LTMG are considered as multi-modality model, and each expression value can be assigned to the model component with the maximal likelihood. For such bimodal or multi-modal models, the *M3S.test* further conducts a consistency test to assess if one sample class is significantly associated with one model component, by using a Fisher Exact test of a two by two contingency table, for each pair of sample condition and model component.
